# Flash Tempering of High-Strength, Low-Alloy Martensitic Steel via Electrical Pulsing Treatment

**DOI:** 10.3390/ma18010182

**Published:** 2025-01-04

**Authors:** Chuhan Zhang, Xi Luo, Jun Liu, Xuefeng Zhou, Yiyou Tu

**Affiliations:** 1 School of Materials Science and Engineering, Southeast University, Nanjing 211189, China; i1239446088@163.com (C.Z.); xuefengzhou@seu.edu.cn (X.Z.); 2 Jiangyin Xingcheng Special Steel Works Co., Ltd., Jiangyin 214400, China; x8102255374@citicsteel.com

**Keywords:** electrical pulsing treatment, fast tempering, residual stress

## Abstract

Optimized heat treatment processes for high-strength, low-alloy steel are studied in order to maximize the strengthening effects of the alloying elements and achieve a favorable balance of strength and ductility. In this study, it is found that high-energy-density electric pulse treatment (EPT) can effectively reduce the residual stress in quenched high-strength, low-alloy steel. Furthermore, EPT promotes the precipitation of fine needle-like ε-carbides and small spherical M_6_C carbides. Using an electrical pulse acting for 10 s with a current density of 19.52 A/mm^2^, the elongation of the quenched steel increases from 4.5% to 12.4%. Compared to the as-quenched state, there is no significant decrease in tensile strength, while the product of strength and ductility reaches 20.33 GPa%. When the pulse current density is increased to 27.76 A/mm^2^ for 10 s, the tempering effect is equivalent to that of tempering at 600 °C for 4 h.

## 1. Introduction

The quest for high-performance materials exhibiting superior mechanical properties has long been central to the field of materials science and engineering. Martensitic steel, renowned for its exceptional strength and ductility, has traditionally been the material of choice for applications requiring high mechanical performance. Over the years, the optimization of its properties through heat treatment—particularly tempering—has been the subject of extensive research. Historically, the investigation of tempering processes for martensitic steel has been a cornerstone of metallurgical research. The pioneering work by Hollomon and Jaffe [1] provided foundational insights into the time–temperature relationships governing the precipitation of lath martensite, thereby laying the groundwork for subsequent studies on the influence of heat treatment on the microstructural evolution and mechanical properties of martensitic steels.

In recent years, research has shifted towards the innovative concept of rapid tempering, often utilizing industrial processes such as induction heating, which offers reduced processing times and energy consumption [2,3,4]. This accelerated tempering process has demonstrated promising results in terms of providing enhanced mechanical properties compared to conventional methods, thereby opening new avenues for industrial applications in which both mechanical performance and production efficiency are critical. In 2018, Judge et al. introduced rapid thermal processing to enhance the toughness of steel, highlighting the potential benefits of rapid tempering in modifying the mechanical properties of steel and clarifying the relationships between the processing conditions and resulting toughness [5,6].

Electric pulse treatment (EPT) has been found to be effective for rapid heat treatment, offering unique advantages such as reducing the processing time and energy consumption [7,8,9,10,11]. We aim to advance this field through investigating the application of high-energy EPT in combination with rapid tempering to optimize the microstructure and mechanical properties of low-alloy martensitic steels. This approach seeks to provide an efficient and energy-saving method facilitating future advancements in terms of material performance and industrial progress.

## 2. Materials and Methods

### 2.1. Materials and Experimental Setup

The chemical composition of the martensitic steel used in this research is detailed in Table 1. Hot-rolled sheets were austenitized at 850 °C for 1 h, then quenched in water. Thereafter, tempering treatments were performed from 200 °C to 600 °C, with the holding time varying from 2 h to 6 h. At the end of holding, the samples were air-cooled.

**Figure 1:**
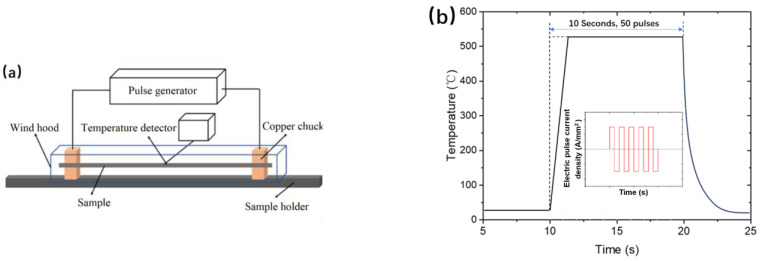
(**a**) Diagram of the electrical pulse treatment processing device; and (**b**) thermal cycle involved in electrical pulse treatment for rapid tempering (27.76 A/mm^2^).

As-quenched steel was cut to a size of 2 × 2 × 140 mm^3^ and its surface was polished using 1500-mesh sandpaper (Shanghai Zhongyan Instrument Manufacturing Factory, Shanghai, China). The process of the EPT is shown in Figure 1. The pulse was supplied by an IT6600C Series Bidirectional Programmable DC Power Supply (ITECH, Nanjing, China), which can reach a maximum power of 42 kW. Figure 1a depicts the device used to apply electric pulses to the samples. The samples were clamped between two chucks made of copper, which were large in size to minimize contact resistance. A thermocouple was welded to the sample in order to monitor the temperature change of the sample in real time. A wind hood was used to reduce external interference and improve the accuracy of the real-time temperature measurements in samples. The pulse current density was set to 16.92, 19.52, 22.48, 25.13, or 27.76 A/mm^2^. The pulse width was 100 μs, and the total action time was 10 s. The peak temperatures of the sample under different pulse current densities were detected using the temperature detector, which were 150, 195, 280, 340, and 545 °C, respectively. Figure 1b shows a schematic diagram of the temperature variation in the sample during the electrical pulse treatment, from which, it can be seen that the sample heated up rapidly when the high-energy-density electrical pulse was applied. Due to the small size of the sample, once the pulse stopped, it quickly cooled to room temperature (i.e., within seconds).

### 2.2. Microstructure Characterization

To observe the microstructural evolution caused by conventional tempering and EPT flash tempering, microstructural characterization of samples was carried out via Nova Nano SEM450 (FEI, Hillsboro, Oregon, USA), operated at 15 kV and Talos F200X TEM (Thermo Fisher Scientific, Prague, Czech Republic), operated at 200 kV. The samples for SEM were etched with 4% nitrate alcohol mixture. Thin foils for TEM were prepared via GATAN-691 Twin-Jet Electropolishing (GATAN, Pleasanton, CA, USA), which was carried out in a mixture of 90% ethanol and 10% perchloric acid at −15 °C with an applied voltage of 35 V.

An electron backscatter diffraction (EBSD) system (EDAX/TSL, Hikari, Hayward, CA, USA) coupled with field emission scanning electron microscopy was used to observe the microstructure. The acceleration voltage and working distance were 15 kV and 15 mm, respectively. Post-processing was performed using TSL OIM Analysis 7 software. The critical misorientation angle for grain identification was set to 15°. The grain size and kernel average misorientation (KAM) were measured to investigate the effect of j on the grain growth and dislocation recovery during EPT. KAM is an indicator of the local grain misorientation and the density of geometrically necessary dislocations for non-recrystallized pixels [12], allowing for the determination of the state of microscopic stress distribution in the material.

### 2.3. Measurement of Mechanical Properties

Ultimate tensile strength (UTS) tests were performed at room temperature with a constant speed of displacement. The initial strain rate was 5.0 × 10^−4^/s. Vickers micro-hardness measurements were performed at 9 locations on the polished surfaces, using a 500 g load and holding time of 10 s.

## 3. Results

### 3.1. Mechanical Properties

**Figure 2:**
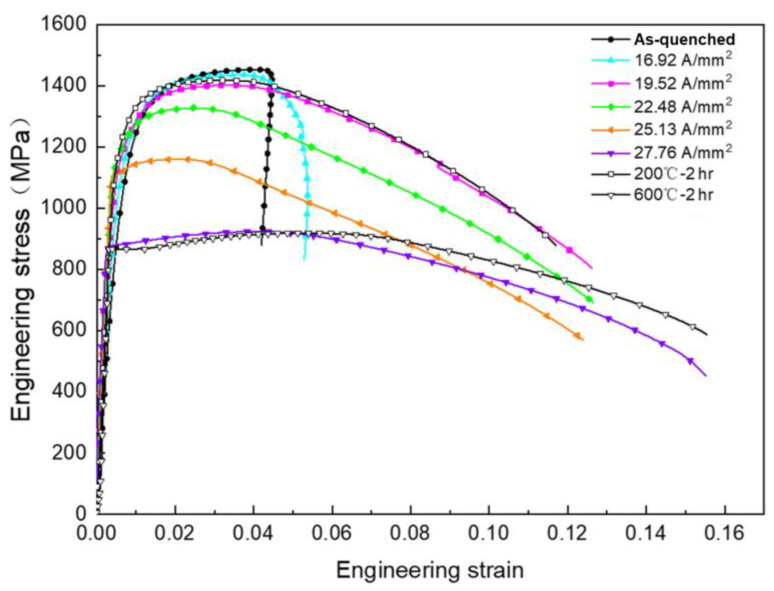
Engineering stress–strain curves of different test samples.

Figure 2 presents the tensile stress–strain curves of the quenched steel plates after being processed using electrical pulses with different current densities. It can be observed from the figure that, after EPT with a current density of 16.92 A/mm^2^, no obvious changes occurred in the mechanical properties of the specimens, and its engineering stress–strain curve was essentially consistent with that of as-quenched specimens. However, when the current density rose to 19.52 A/mm^2^, the tensile strength of the specimens decreased by 50 MPa and the elongation sharply increased to 12.4%, which was nearly twice that of the as-quenched samples. The product of strength and ductility is a comprehensive performance indicator characterizing the level of toughness of metallic materials. It is the product of the tensile strength (σ_b_) of steel and its elongation after fracture (δ). The product of strength and elongation increased from 6.53 GPa% to 20.33 GPa%, significantly improving the toughness of the steel plates. A distinct necking zone emerged on the tensile fracture surface, indicating a significant improvement in the plasticity of the steel plates. As the pulsed current density further increased to 22.48 A/mm^2^, the tensile strength of the steel plates decreased significantly to 1327 MPa, but the elongation was comparable to that of the specimens processed with a current density of 19.52 A/mm^2^; as the current density continued to increase, after being processed with a 27.76 A/mm^2^ pulsed current density, the engineering stress–strain curve of the specimens began to exhibit a yield plateau, and its tensile strength was greatly reduced to only 924 MPa, which was equivalent to that of the specimens in the tempering state at a high temperature of 600 °C.

Comparing the mechanical properties of the specimens processed via conventional tempering treatments, it can be noted that those of the sample treated with 10 s EPT at a current density of 19.52 A/mm^2^ essentially overlapped with the tensile curve of the specimens tempered at 200 °C for 2 h, presenting similar mechanical properties. The effect of 10 s EPT with a current density of 27.76 A/mm^2^ was basically consistent with that of tempering at 600 °C for 2 h. Thus, it can be concluded that EPT, as an efficient tempering technique for quenched steel plates, not only significantly enhances the strength and toughness of steel plates but also demonstrates extremely high efficiency and holds great potential.

### 3.2. Microstructure Characterization

**Figure 3:**
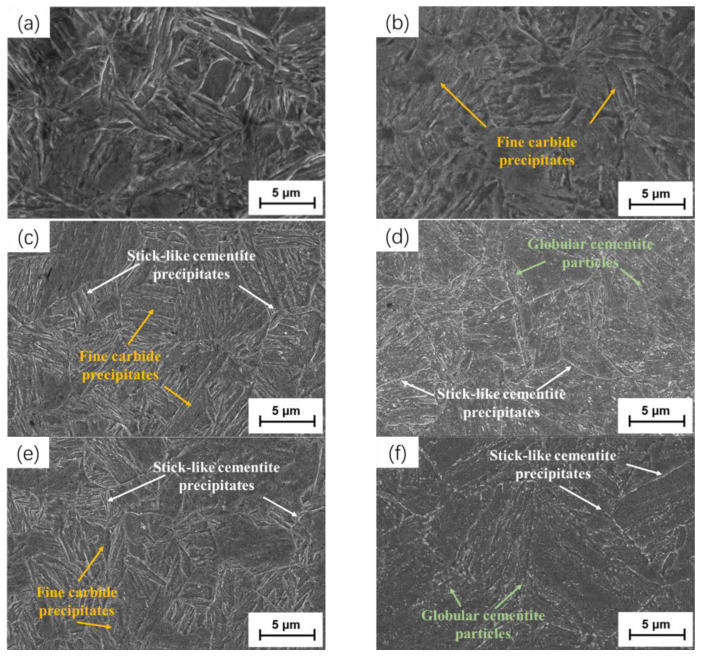
SEM images of (**a**) as-quenched sample; samples after EPT at (**b**) 16.92 A/mm^2^, (**c**) 19.52 A/mm^2^, and (**d**) 27.76 A/mm^2^; and samples after traditional tempering at (**e**) 200 °C for 2 h and (**f**) 600 °C for 2 h.

Figure 3 depicts the microstructural morphology observed under a scanning electron microscope of the quenched steel plate samples after electrical pulse treatment and tempering at different temperatures. Figure 3a represents the microstructure of the as-quenched steel plate, from which, it can be discerned that the microstructure of the steel plate after quenching was dominated by lath martensite, with diverse orientations of the lath bundles. Figure 3b indicates that, after treatment with pulse current densities of 16.92 A/mm^2^, there was no conspicuous change in the microstructure of the steel plate, which remained a lath martensite structure. At this point, the electrical pulse treatment had a minor influence on the microstructure of the steel. When the pulse current density increased to 19.52 A/mm^2^, the martensite laths in the steel plate remained intact, with no obvious alterations, but a high density of carbides precipitated in the laths, as illustrated in Figure 3c. When the current density further increased to 27.76 A/mm^2^, real-time temperature detection indicated that the peak temperature in the sample reached 543 °C, and the martensitic platelets were completely decomposed (Figure 3d); as such, the tensile strength of samples dropped significantly (to 924 MPa), while the plasticity significantly improved (with an elongation rate of >15%).

In comparison with the sample after low-temperature tempering at 200 °C for 2 h, the lath martensite still retained the lath morphology (Figure 3e). Carbides precipitated from the lath martensite, leading to a decrease in the tetragonality of the martensite, a reduction in internal stress, and a significant improvement in plasticity, compared to the quenched state. At this point, due to the maintenance of the lath martensite morphology, a large number of carbon atoms were still segregated near the dislocation lines, and the tensile strength of the sample steel only decreased by approximately 40 MPa, compared to the quenched state. When high-temperature tempering was carried out at 600 °C, most of the lath martensite decomposed, and a large number of film-shaped carbides precipitated along the original austenite grain boundaries and within the laths. The internal dislocation density decreased, and the precipitated cementite began to agglomerate (Figure 3f); in particular, at this time, as the lath morphology could not be maintained and the agglomerated cementite began to grow and spheroidize, the tensile strength of the sample steel showed a significant decrease (of approximately 37%), compared to the as-quenched state. Meanwhile, due to the occurrence of recovery and recrystallization within the sample, the internal stress was reduced and the plasticity significantly improved.

**Figure 4:**
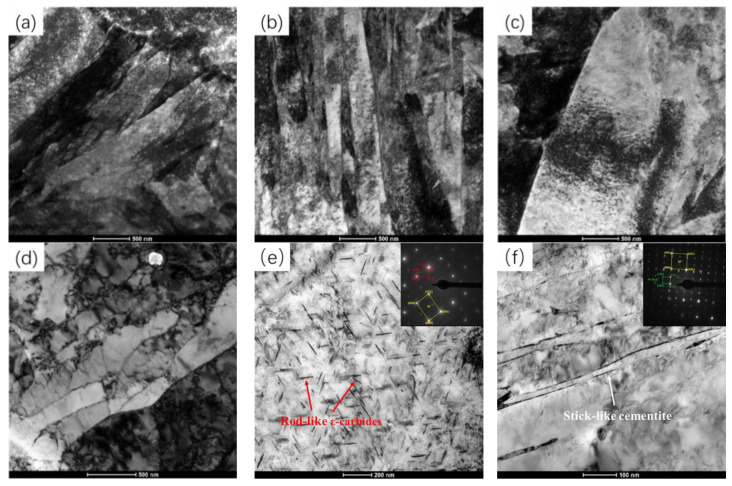
TEM images of (**a**) as-quenched sample; samples after EPT at (**b**) 12.03, (**c**) 19.52, and (**d**) 27.76 A/mm^2^; and the precipitation (carbides) in the samples after EPT at (**e**) 19.52 and (**f**) 27.76 A/mm^2^.

Figure 4a presents the transmission electron microscope (TEM) image of the quenched sample, while Figure 4b–d represent the TEM images after electrical pulse treatments with current densities of 16.92, 19.52, and 27.76 A/mm^2^, respectively. It can be seen from Figure 4a that in the as-quenched sample, the width of the martensite laths was approximately 500 nm, and there was a high density of internal dislocations. A large number of dislocations were mutually intertwined and clustered, densely distributed within the laths and at the lath boundaries. This indicates that, after water quenching, the dislocations in the sample steel rapidly multiplied and formed a high-density entangled dislocation configuration due to the interactions between dislocations. Figure 4b presents the microstructural and dislocation configuration of the specimen after being treated with a 16.92 A/mm^2^ electrical pulse. It can be observed from the figure that, under the influence of the pulsed current, the high-density dislocation configuration within the martensitic laths was adjusted, resulting in the formation of a large number of bundled lamellae, with the lamellar thickness in the range of 100 to 200 nm. Figure 4c shows that, after the electrical pulse treatment with a current density of 19.52 A/mm^2^, the entanglement between dislocations within the martensite lath lamellae weakened, the free path of dislocations increased, and the plasticity of the steel plate was significantly improved. With the pulse current density increased to 27.76 A/mm^2^, the microstructure of the sample after electrical pulse treatment is shown in Figure 4d, from which, it can be seen that recrystallization and recovery occurred in some areas of the sample, forming a blocky ferrite structure. The morphology of the lath martensite was completely decomposed, a large number of dislocations disappeared and were annihilated, and fine-striped carbides precipitated at the lath boundaries. This indicates that, due to the excessive current density, the instantaneous measured temperature of the sample was 543 °C, reaching the recrystallization temperature. Under the combined action of the thermal and non-thermal effects of the high-energy electrical pulse, the decomposition of the lath martensite was accelerated, and the migration of atoms and precipitation of the second phase were promoted.

Electric pulse treatment, through the coupling of Joule heating and electron wind force effects, reduces the diffusion activation energy of carbon atoms in martensitic steel. This increases their diffusion rate, promoting dissolution from the martensitic lattice and the formation of ε-carbides and Fe_3_C [13,14]. Figure 4e shows the TEM images and selected area electron diffraction (SAED) of the carbides in the sample after EPT with a pulse current density of 19.52 A/mm^2^. It can be observed that, under this pulse current density, a high density of short rodlike carbides precipitated in the martensitic laths. The widths of the carbides were in the range of 10–25 nm, with lengths ranging from 50 to 220 nm. The acicular precipitated phase was identified as ε-carbides. The formation of these carbides consumed carbon in the martensite matrix, reducing the solid solution strengthening of the martensitic matrix. However, due to the favorable precipitation strengthening effect, the strength of the sample did not change significantly. Figure 4f shows the TEM images and selected area electron diffraction of the carbides in the sample after EPT at 27.76 A/mm^2^ for 10 s. It can be seen that, under the action of the electrical pulse with this current density, large quantities of stick-like Fe_3_C precipitated along the martensite plate boundaries.

## 4. Discussion

Considering the influence of both temperature and time on the tempering process, the tempering cycle is typically summarized based on the widely used Hollomon and Jaffe (HJ) parameters [1,15], which effectively represent the hardness response during tempering. The HJ parameters are calculated using the following formula:

(1) $$P=T\left[c+\log\left(t\right)\right].$$

The HJ tempering parameter, denoted by *P*, is incorporated in the equation. The temperature (K) is represented by *T*, while the material constant (13 for the specific material utilized in this study [1]) is denoted by *c*. Additionally, *t* signifies the holding time (seconds).

**Figure 5:**
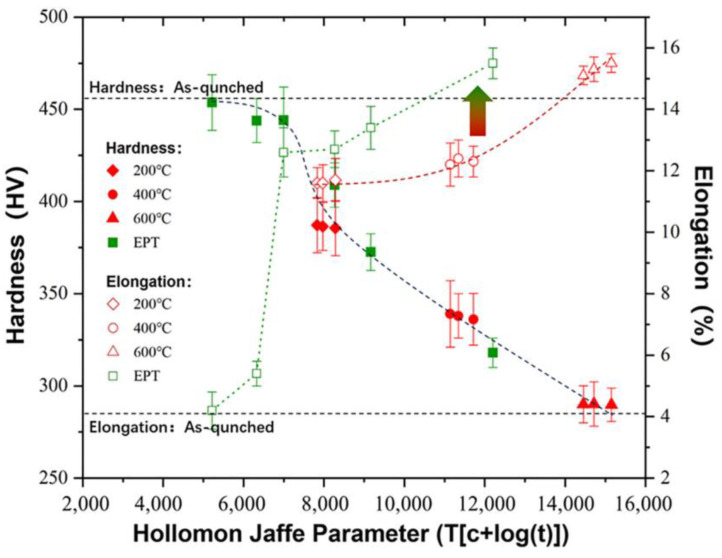
The relationships between the H-J parameter, microhardness, and elongation of the samples.

The value of *P* was calculated by substituting the real-time collected sample temperatures and processing times into the aforementioned formula. Figure 5 presents a plot depicting the relationship between the HJ tempering parameter, microhardness, and elongation of the samples. The graphical representation reveals that the application of EPT induces tempering softening of the steel plate, as evidenced by an increase in the pulse current (leading to an elevated *p* value).

When *P* > 7000, the hardness of the steel plate dropped sharply, entering the near-linear softening stage. From the variation pattern of the steel plate elongation, it can be seen that, under a similar degree of softening, the electric pulse-treated steel plate exhibited superior plasticity. Particularly, when *P* ≈ 8000 (8264: tempered at 200 °C for 6 h; 8268: EPT at 22.48 A/mm^2^ for 10 s), the hardness of the EPT steel plate was 23 HV (6%) higher than that of the tempered steel plate, while the elongation was also 10% higher. EPT demonstrated excellent performance in terms of obtaining high-strength and high-toughness steel plates. Among them, the steel plate treated with an electric pulse of 19.52 A/mm^2^ for 10 s had a strength and hardness that basically matched the values of the as-quenched steel plate, while its elongation was three times that of the as-quenched steel plate. The figure also demonstrates that the hardness of the tempered steel sample, when subjected to tempering temperatures ranging from 200 to 600 °C, was predominantly influenced by the tempering temperature and remained essentially unaffected by the duration of annealing (2–6 h). A similar rule was also discovered for the EPT samples, where the influence of the pulse processing time (pulse number) on the mechanical properties of the steel plate was minor, while the pulse current density was the decisive factor.

To investigate the factors contributing to the steel plate’s ability to maintain high strength and achieve significant enhancement in plasticity under a pulse current density of 19.52 A/mm^2^, an orientation difference analysis was conducted using EBSD. The average orientation difference (KAM) provides insights into the average dislocation density/strain distribution within a sample, while the local orientation difference effectively characterizes localized strain and, to some extent, reflects the degree of stress concentration [16].

**Figure 6:**
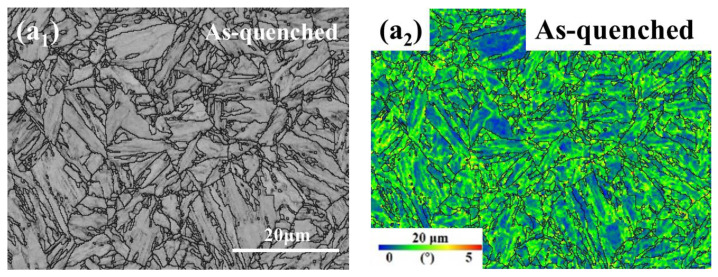

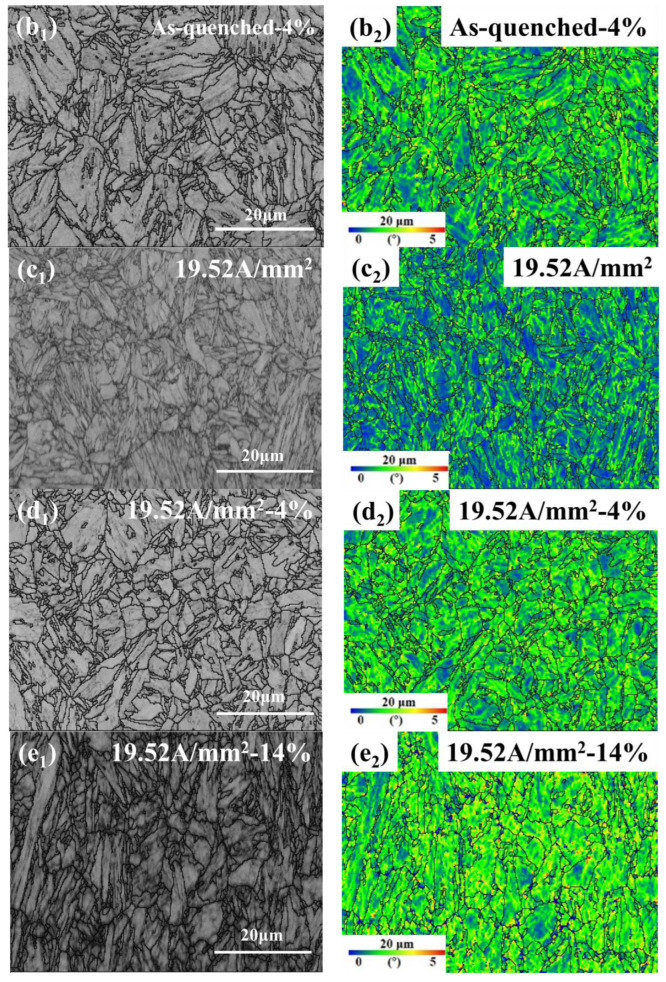

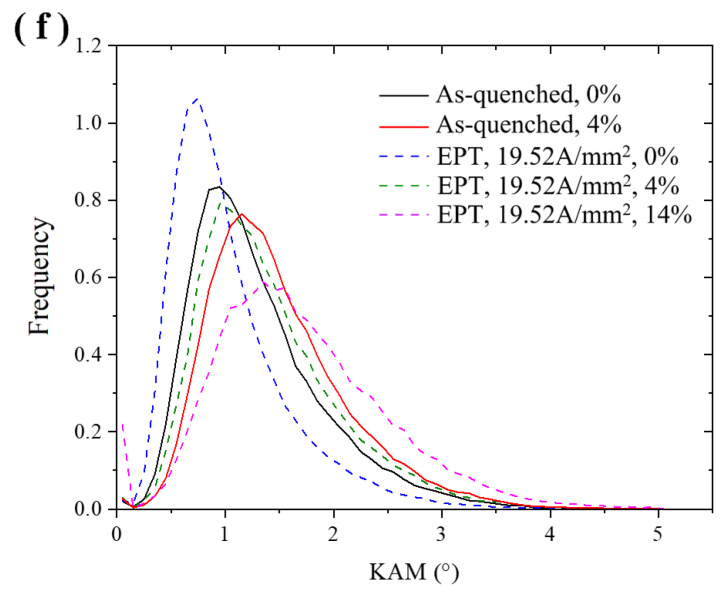
(**a1**) Backscattered electron SEM images and (**a2**) KAM maps of the as-quenched sample; (**b1**,**b2**) after 4% tensile deformation; (**c1**,**c2**) sample after EPT at 19.52 A/mm^2^; (**d1**,**d2**) after 4% tensile deformation; (**e1**,**e2**) the necking region after 14% tensile deformation; and (**f**) kernel average misorientation frequency of the samples.

The EBSD analysis was conducted to investigate the strain distribution in the as-quenched and EPT samples after undergoing different tensile deformations, as illustrated in Figure 6. The KAM of the quenched sample was approximately 1.33°, as evident from Figure 6(a1,a2 and f), revealing numerous regions with high-density dislocations and relatively elevated residual stress. The volume expansion resulting from the martensitic phase transformation caused by rapid cooling induced lattice distortion, leading to the observed phenomenon. Comparing Figure 6(a1,a2,c1,c2), it is evident that the EPT effectively reduced the dislocation density and residual stress while significantly increasing the blue low-strain region in the figure. This is further supported by a decrease in KAM to 1.03°, as shown in Figure 6f.

The above results indicate that the residual stress in the specimens was significantly reduced through EPT. Generally, plastic deformation is closely associated with the generation and movement of dislocations [17]. The decrease in dislocation density and the rearrangement of the dislocation structure with the use of electropulsing have been reported widely [7,10,17,18,19,20,21]. Using the concept of thermally activated plastic flow, it was found that drift electrons affect thermally activated dislocation motion in addition to exerting force on dislocations via electron wind. Drift electrons enhance the movement of vacancies and dislocations, accelerating the annihilation of dislocations [7]. During mechanical deformation, electropulsing enhances cross-slip and twinning, producing a wavy dislocation morphology similar to cryogenic deformation. This prevents dislocations from localizing into planar slip bands, which would otherwise lead to early tensile failure of the alloy [21].

After undergoing a small strain (4%) tensile deformation, a significant number of high-strain regions (orange–red) emerged at the lath boundaries and grain boundaries of the as-quenched sample (refer to Figure 6(b2)). However, there was only a marginal change in the KAM value representing the overall uniform deformation, with an observed increase from 1.33° to 1.49° (refer to Figure 6f). This suggests that the quenched steel plate exhibited poor overall uniform deformation during tensile deformation, with strain concentration occurring at positions such as martensite lath boundaries and grain boundaries. The local strain concentration led to the premature initiation of cracks and fractures, resulting in low overall elongation.

The dislocations in the lath martensite significantly increased in the sample treated with electric pulse processing, reaching a level comparable to that of the as-quenched sample after 4% strain deformation. This was evidenced by an increase in the KAM value from 1.03° to 1.40° (see Figure 6(d1,d2,f)). Additionally, based on the directional difference distribution results, it can be observed that the electric pulse-processed sample exhibited a lower degree of maximum local orientation difference after undergoing 4% deformation, indicating excellent deformation uniformity. When the tensile strain was further increased to 14%, the sample presented a distinct necking phenomenon. The EBSD results revealed that the KAM value in the necking region reached as high as 1.69°, indicating that intense plastic deformation occurred within the material at this time and the dislocation density was very high. Nevertheless, as a consequence of the combined impact of the thermal and non-thermal effects associated with the electrical pulse treatment, a substantial number of mobile dislocations were produced in the sample before deformation. This phenomenon enhanced the homogeneity of strain distribution, mitigated stress concentration, postponed the strain concentration at sites such as grain boundaries, ameliorated the uniform rheological capability of the material, and augmented its plasticity.

## 5. Conclusions

The influence of electrical pulse treatments with various current densities on the mechanical properties and microstructure of high-strength, low-alloy steel was investigated. The effects of the electrical pulses were analyzed, and a comparison was made with samples prepared via traditional quenching–tempering treatment. The main conclusions are as follows:

When subjected to an electrical pulse with a current density of 19.52 A/mm^2^ for a duration of 10 s, the quenched high-strength, low-alloy, hot-rolled steel exhibited an increase in elongation from 4.5% to 12.4%. Furthermore, compared to its quenched state, there was no significant reduction observed in its tensile strength, resulting in a product of strength and ductility reaching up to 20.33 GPa%. When the pulse current density was increased to 27.76 A/mm^2^ for a duration of 10 s, the tensile strength of the quenched high-strength, low-alloy steel plate presented a notable decrease accompanied by the emergence of a yield plateau; however, it achieved an elongation value of 15.5%, equivalent to annealing at 600 °C for 4 h. Overall, the tempering treatment using high-energy density electrical pulses led to remarkable efficacy and efficiency.

After the high-strength, low-alloy, hot-rolled steel was quenched and subjected to a pulsed current of 19.52 A/mm^2^ for 10 s, the martensite lath in the steel plate remained intact and exhibited a relatively high dislocation density. The application of electrical pulses resulted in the liberation of numerous dislocations from their constraints, leading to the reorganization of dislocations within the lath and the formation of substantial lamellar substructures with widths ranging from 100 to 200 nm. This process weakened the entanglement effect between dislocations within the lamellae, increased their free path, and transformed them into mobile dislocations, thereby significantly enhancing the plasticity of the steel plate. Additionally, a high number of fine needle-like ε-carbides precipitated between the laths, contributing significantly to precipitation strengthening.

Electrical pulses were demonstrated to effectively mitigate the residual stress and dislocation density in quenched high-strength, low-alloy, hot-rolled steel. Specifically, when subjected to an electrical pulse of 19.52 A/mm^2^ for a duration of 10 s, the average orientation difference (KAM) decreased from 1.33° to 1.03°. The KAM analysis revealed that, during tensile deformation of the quenched steel plate, the overall uniform deformation was relatively limited, with the strain concentration primarily occurring at critical locations such as martensite lath boundaries and grain boundaries. The intense concentration of strain resulted in the premature initiation of cracks and subsequent fracture. In the samples subjected to electrical pulses, a significant proliferation of dislocations in the lath martensite was observed after 4% tensile deformation, demonstrating exceptional deformation uniformity. This enhances the material’s overall rheological capacity and improves its plasticity.

Flash tempering of martensitic steel via electrical pulsing treatment, involving shorter times and faster heating rates than conventional tempering, offers an improved combination of tensile properties and fracture toughness, reduced processing times, and lower energy costs. A properly designed electrical pulsing treatment could be considered as a suitable alternative to traditional heat treatment.

## Figures and Tables

**Table 1:** Chemical composition of the steel in this research (wt.%).

Fe	C	Ni	Mn	Cr	Mo	Si	V	Nb
Bal.	0.179	1.30	0.79	0.47	0.37	0.21	0.016	0.011

## Data Availability

The original contributions presented in this study are included in the article. Further inquiries can be directed to the corresponding author.
